# Familial Multiple Sclerosis in a Mother and Son Pair: A Sri Lankan and a South Asian First

**DOI:** 10.1155/2021/1172870

**Published:** 2021-09-23

**Authors:** Ishani Rajapakshe, Bimsara Senanayake

**Affiliations:** Institute of Neurology, National Hospital of Sri Lanka, Colombo, Sri Lanka

## Abstract

Multiple sclerosis (MS) is an immune-mediated demyelinating disorder involving the central nervous system (CNS). It is common amongst young females. Although the exact cause of MS is yet unknown, viral infections such as EBV, environmental factors, and autoimmune and genetic mechanisms involving HLA-DRB1 loci are implicated. Familial MS is reported from some geographic locations and ethnic groups but is thought to be rare in Asia. In this paper, we present both a Sri Lankan mother and her son, with clinically definite MS conforming to McDonald's 2017 clinical and MAGNIMS 2016 radiological criteria. Both had oligoclonal bands in their CSF (OCB-IEF) with no serum bands indicating intrathecal production and were negative for AQP4 and MOG IgG serology. Familial MS is more common among siblings, with sister-sister relationship having the highest rate. The lowest relation was amongst father-son and mother-son pairs. Amongst siblings, the risk of MS is between 3.5% and 4.7%. Inherited factors rather than common environmental exposure influence susceptibility in such cases. To the best of our knowledge, MS occurring in a mother-son pair has not been reported before either from Sri Lanka or South Asia.

## 1. Introduction

MS is an immune-mediated neurological disorder affecting the central nervous system (CNS) with no clear cause. Pathologically, it demonstrates neuroinflammation, demyelination, remyelination, neurodegeneration, and astrogliosis due to immune-mediated destruction of neurons. MS occurs in either relapsing or progressive forms. Possible etiological factors are immune, genetic, and environmental, and mostly MS occurs sporadically [1, 2]. MS is known to accumulate within families, but until the 1980s, familial MS was thought to be “quite exceptional.” However, some believed it to be an “inherited transmissible” disease. MS is diagnosed using the modified McDonald criteria of 2010 and 2017 and 2016 MAGNIMS MRI criteria [3]. The risk of familial MS is 12- to 38-fold in siblings, 6- to 25-fold in children of MS patients, and 7- to 26-fold in parents [2]. Familial MS is common in high MS prevalent areas but is very rare in Asia. Only a few reports are available on familial MS from low prevalent regions. We present a pair of clinically definite patients with MS who happen to be mother and son.

## 2. Case Report

### 2.1. Son

**Figure 1:**
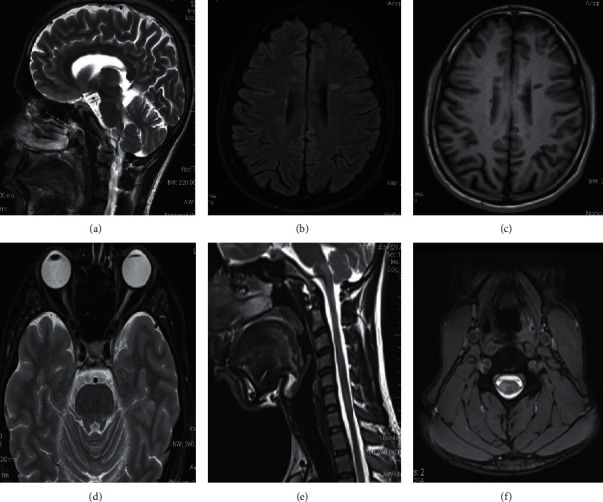
MRI brain and spine of son: (a) T2 sagittal, (b) T2 FLAIR axial, (c) T1 axial images demonstrate numerous T2 high signal T1 low signal white matter lesions arranged perpendicular to the bilateral lateral ventricles suggesting Dawson's fingers. Lesions involve the corpus callosum, callasoseptal interface, and posterior fossa. T2 axial image of the orbits (d) shows right optic neuritis. Cervical spine T2 sagittal (e) and axial (f) images demonstrate multiple short segment T2 high signal lesions without significant spinal cord edema and expansion. Lesions are not contrast enhancing.

**Figure 2:**
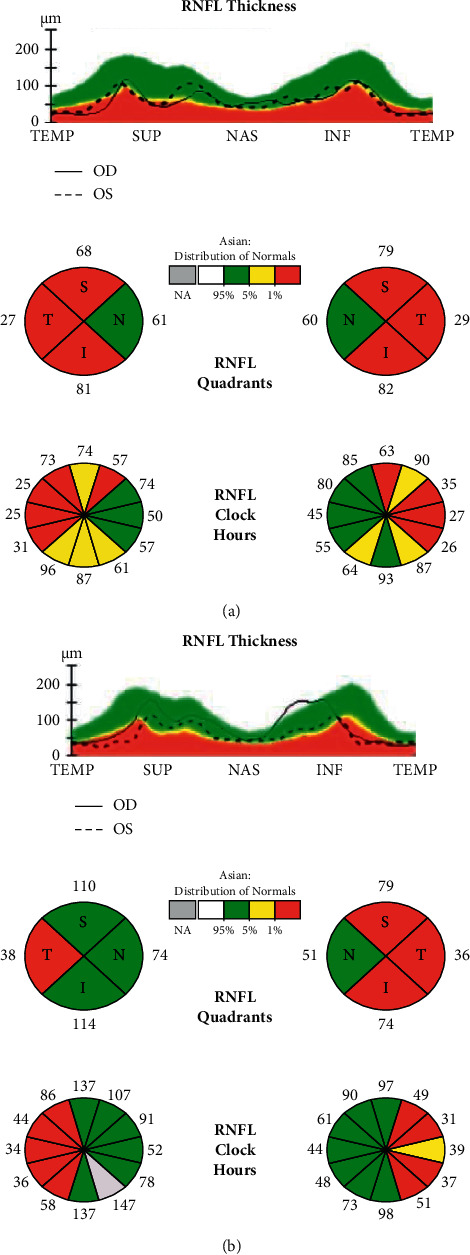
Optical coherence tomography shows retinal nerve fiber layer of optic nerve heads. (a) Mother: bilateral severely affected retinal nerve fiber layer. (b) Son: severely affected superior, inferior, and temporal quadrants of the left eye and severely affected temporal quadrant of the right eye.

A 26-year-old South Asian male presented with progressive unsteadiness and difficulty walking for 1 year. Initially, he noticed weakness of his right lower limb which partially recovered spontaneously and then progressed to affect the left lower limb. He also developed left-sided painful visual loss after 6 months which resolved with some residual deficiency. The upper limbs were spared, and sensations were intact. Examination revealed spastic paraparesis without sensory involvement and left-sided cerebellar signs. Left-sided blurred disc margin with relative afferent pupillary defect (RAPD) was also noted indicating an anterior visual pathway lesion. There was bladder involvement with features suggestive of voiding dysfunction. His EDSS was 3.5 and cognition was normal. MRI brain (Figure 1) showed multiple discrete T2-weighted and flair hyperintensities in periventricular, juxtacortical, and infratentorial regions with and without gadolinium enhancement. MRI spine showed multiple short segment lesions which are more confluent. CSF for oligoclonal bands was positive, and visual evoked potentials (VEP) showed a left-sided delay. His OCT (Figure 2) showed bilateral temporal visual field retinal nerve fiber layer (RNFL) thinning. He fulfilled both MAGNIMS 2016 MRI criteria and McDonald 2017 criteria for the diagnosis of MS. Diagnosis of primary progressive MS was made.

### 2.2. Mother

**Figure 3:**
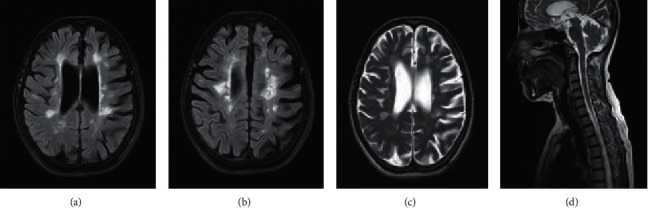
T2 FLAIR axial (a, b) and T2 axial (c) show multiple periventricular Dawson's fingers and juxtacortical lesions. Significant cerebral atrophy is seen with enlarged lateral ventricles suggesting chronic disease. T2 sagittal images of the cervical and upper dorsal spine (d) show multiple short segment lesions without significant cord expansion.

At the age of 43, she developed weakness of both lower limbs, gait instability, and dysarthria which resolved partially and similar episode with visual impairment after 2 years for which Ayurveda medicine was taken. Then, her disability progressed over the next 8 years. After an initial assessment at a neurology unit, she sought native treatment. Her EDSS was 7.0 and cognition was severely affected with an MoCA of 6/30. She has had evidence of both voiding and storage dysfunction. Her bowel functions were normal. Neurological exam revealed spastic paraparesis with bilaterally impaired joint position sense without a sensory level, bilateral cerebellar signs, and left-sided RAPD. Neuroimaging (Figure 3) was suggestive of MS, and CSF-OCB was positive. VEP was absent in her left side, and OCT showed bilateral RNFL thinning (Figure 2). She fulfilled both McDonald 2017 and MAGNIMS 2016 MRI criteria for a diagnosis of MS. Diagnosis of primary progressive MS was made.

Except for the mother and son, other family members have not shown similar neurological manifestations. Both were of Sri Lankan ancestry, and there was no history of foreign travel. Summary of clinical details is provided in Table 1.

## 3. Discussion

Multiple sclerosis (MS) is an immune-mediated CNS demyelinating disorder without a clear cause. It involves the brain, cerebellum, spinal cord, and optic nerve. MS is thought to be the most common disabling neurological disease of the CNS in the West with a global prevalence of 33 per 100,000 [1]. However, in Asia and Africa, the prevalence is relatively low demonstrating a striking geographic variation. Worldwide, women are more likely to suffer from MS than men in a ratio of 2:1. Epidemiological data from South Asia on MS and related disorders are limited. However, a steady increase in the number of cases diagnosed with MS in this region has been seen recently. This is probably due to greater awareness, better imaging facilities, and the expansion of neurological services in this part of the world. Available limited data estimate the prevalence of MS in Asia to be between 0 and 20 per 100,000 [2, 3]. Compared to the rest of the world which has a female to male ratio of 2 : 1 for MS, in Asia, it is 3.6 : 1 [4]. In Sri Lanka, too accurate epidemiological data on MS are sparse [5, 6]. However, according to hospital-based demographic studies conducted, the female to male ratio is 1.7 : 1 with the commonest type of MS being RRMS [7]. Most patients demonstrate a good outcome with a low disability progression [8]. Early detection and wider use of disease-modifying drugs could be a reason for this [8]. The EDSS in comparison was slightly higher in males in Sri Lankan patients, 3.01 vs. 2.6. The annualized relapse rate (ARR) of 0.49 per year was noted in those cases with a median disease duration of 7.9 years. None of these descriptive demographic studies have identified any familial MS cases in Sri Lanka prior to this. In our extensive search, we could not find any such mother-son pairs in South Asia either. In this case, the son is a product of nonconsanguineous Sri Lankan parents. The clinical presentation and radiological evidence were similar in both the mother and the son. Both had oligoclonal bands in the CSF (OCB-IEF) with no serum bands. The abnormalities seen on visual evoked potential (VEP) testing and optical coherence tomography (OCT) in both patients supported a diagnosis of MS. Both mother and son had relapsing progressive disease and were seronegative for cell-based analysis of AQP4-IgG and MOG-IgG.

The earliest report of familial MS was by Curtius. There are over 100 polymorphisms associated with MS in various studies, and the strongest association is related to certain class I and class II alleles of the MHC, particularly the HLA-DRB1 locus. Mounting evidence suggests that the risk of MS is associated with multiple non-MHC susceptibility genes as well (e.g., CD6, CLEC16A, IL2RA, IL7R, IRF8, and TNFRSF1A). In addition, polymorphisms in the IL-7R gene may slightly increase the risk [9]. Familial MS is rarely reported from Asia. A systematic review shows only 5% patients with MS having a positive family history [9]. Familial MS is commoner in first-degree relatives who run risk of 20–40 times of getting the disease. Mother-child pairs with MS are less common than predicted and father-child pairs with MS were more common than predicted according to observational studies. According to a large population-based cohort study in Saskatoon, the Saskatchewan rate of familial MS was 17.3%, but out of 86 familial MS cases, only 3 mother-son pairs have been recognized [10]. Even in high MS prevalent countries, only a limited number of mother-son pairs have been reported to date. In South Asia, we believe ours is the first such pair.

## Figures and Tables

**Table 1:** Summary of investigations.

	Son	Mother
MRI brain	>3 periventricular lesions and juxtacortical lesions	>3 periventricular lesions and juxtacortical lesions
MRI spine	Multiple spinal plaques	Multiple spinal plaques
McDonald criteria (2017)	Satisfied	Satisfied
MAGNIMS criteria (2016)	Satisfied	Satisfied
VEP	Unilateral delay	Unilateral absent
CSF-OCB (IEF method)	Positive	Positive
Anti-aquaporin-4 (anti-AQP4) and anti-myelin oligodendrocyte glycoprotein (anti-MOG) antibodies cell-based assay (Mayo Clinic, USA)	Negative	Negative
